# *Mentha* Essential Oils: Unraveling Chemotype-Dependent Biosynthesis and Assessing Evidence for Health-Promoting Activities

**DOI:** 10.3390/nu17203258

**Published:** 2025-10-16

**Authors:** Yifan Yu, Yalin Ma, Zhen Ouyang, Luqi Huang

**Affiliations:** 1 School of Pharmacy, Jiangsu University, Zhenjiang 212013, China; yifan_yu@ujs.edu.cn (Y.Y.); 2212315032@stmail.ujs.edu.cn (Y.M.); 2 State Key Laboratory for Quality Ensurance and Sustainable Use of Dao-di Herbs, Beijing 100700, China

**Keywords:** *Mentha* essential oils, chemotypic variation, terpenoid biosynthesis, health-promoting activities, pharmacological applications

## Abstract

*Mentha* essential oils (EOs), renowned for their distinctive aromas and diverse biological activities, represent a key focus in phytochemical and pharmacological research. While numerous reviews have documented the general properties of mint EOs, a systematic and critical synthesis of recent advances linking chemotypic diversity to biosynthetic mechanisms and evidence-based health benefits remains lacking. This review aims to address this gap by comprehensively analyzing the structural variability of EOs across major *Mentha* species, elucidating the chemotype-dependent enzymatic and genetic regulation within the plastidial methylerythritol phosphate (MEP) pathway, and evaluating preclinical and clinical evidence supporting their health-promoting activities, including antimicrobial, antioxidant, anti-inflammatory, digestive, respiratory, cognitive-enhancing, and anticancer effects. By integrating findings from cutting-edge transcriptomic and genomic studies, we highlight how genetic variations and epigenetic factors influence monoterpene biosynthesis and ultimately shape bioactivity profiles. Furthermore, we critically assess challenges related to EO standardization, bioavailability, and clinical translation, and propose interdisciplinary strategies, such as metabolic engineering, nano-delivery systems, and structured clinical trial designs to overcome these barriers. This review not only consolidates the current understanding of *Mentha* Eos, but also provides a forward-looking perspective on their potential applications in functional foods, pharmaceuticals, and personalized health products.

## 1. Introduction

**Figure 1:**
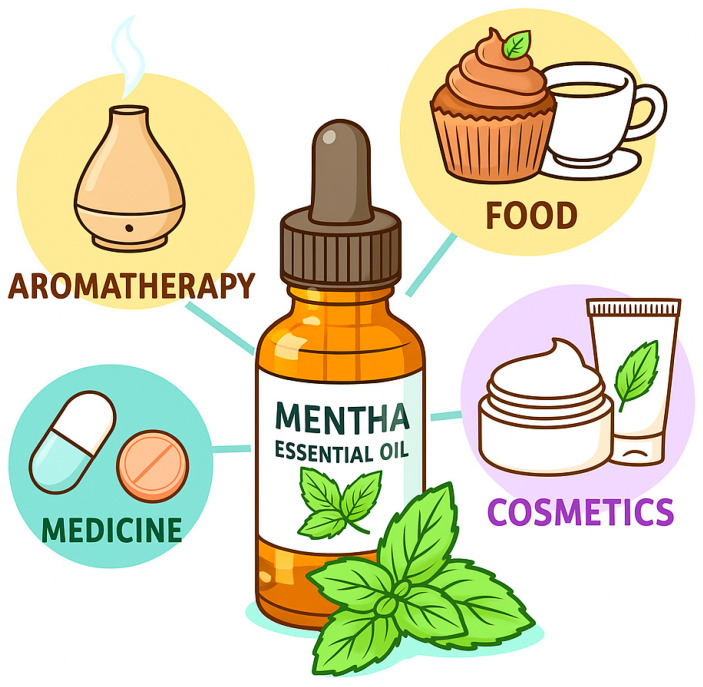
Applications of *Mentha* EOs in Food, Pharmaceuticals, Cosmetics, and Aromatherapy.

The genus *Mentha*, a prominent member of the Lamiaceae family, encompasses a diverse group of aromatic plants widely recognized for their characteristic fragrances and extensive applications in culinary, cosmetic, and medicinal fields [1,2,3] (Figure 1). These applications are primarily attributed to the complex mixtures of volatile organic compounds, predominantly terpenoids, stored within specialized glandular trichomes on the plant surface, collectively known as EOs [4,5]. The distinctive aroma profiles and therapeutic properties of *Mentha* EOs, often referred to as mint EOs, have positioned them as subjects of intense scientific scrutiny, driving research into their chemical composition, biosynthetic origins, and pharmacological potential [6]. The global market for *Mentha* EOs continues to expand, fueled by consumer demand for natural products and a growing appreciation for their health-promoting attributes, ranging from traditional uses in digestive aids and pain relief to emerging applications in antimicrobial and anticancer therapies [7,8,9].

A fundamental aspect of *Mentha* EOs is their remarkable chemical diversity, which is not merely species-specific but often manifests as distinct “chemotypes” within the same species. These chemotypes are characterized by the predominance of particular monoterpenoids, such as menthol, menthone, pulegone, carvone, or limonene, which dictate the oil’s sensory qualities and biological activities [10,11,12,13]. Understanding the factors that govern this chemotypic variation—including genetic predispositions, environmental influences like climate and soil conditions, and even post-harvest processing techniques—is crucial for optimizing the production of EOs with desired chemical profiles and maximizing their therapeutic efficacy [14,15,16]. The intricate interplay between these factors underscores the complexity inherent in harnessing the full potential of *Mentha* EOs for various industrial applications.

Beyond their chemical characterization, the elucidation of the biosynthetic pathways responsible for the production of these valuable monoterpenes has been a significant area of advancement [17,18,19]. The plastidial methylerythritol phosphate (MEP) pathway serves as the foundational route for monoterpene synthesis in *Mentha* species, involving a cascade of enzymatic reactions meticulously regulated at genetic and epigenetic levels [8,20,21]. Advances in genomic and transcriptomic analyses, coupled with metabolic engineering efforts, have begun to unravel the precise enzymatic steps and regulatory mechanisms that lead to the accumulation of specific monoterpenoids, offering promising avenues for enhancing EO yield and tailoring their composition through biotechnological interventions [17,22]. Such detailed understanding is pivotal for developing sustainable and efficient production strategies that meet the increasing demand for high-quality *Mentha* EOs.

The health-promoting activities of *Mentha* EOs are extensive and well-documented, spanning a wide array of biological effects that are increasingly supported by scientific evidence. These include potent antimicrobial and antifungal properties, making them valuable candidates for combating drug-resistant pathogens and preserving food products [23,24,25]. Their significant antioxidant and anti-inflammatory capacities contribute to their potential in mitigating oxidative stress and chronic inflammatory conditions [5,26,27]. Furthermore, research has highlighted their benefits in neurological and digestive health, offering relief from various ailments, and exploring their potential in respiratory support and cognitive enhancement [28,29]. More recently, the emerging field of anticancer research has begun to investigate the cytotoxic potential of *Mentha* EOs against various cancer cell lines [30,31,32], suggesting novel therapeutic avenues. Despite the compelling biological activities revealed through in vitro and in vivo studies, significant challenges remain in translating these findings into standardized, widely adopted therapeutic products. Key hurdles include ensuring consistent quality and chemotypic stability, overcoming limitations of volatility and poor bioavailability, and conducting rigorous safety assessments alongside well-designed clinical trials. Addressing these translational gaps requires concerted, interdisciplinary efforts spanning phytochemistry, pharmacology, pharmaceutical technology, and clinical research.

While earlier reviews have documented the general properties of *Mentha* EOs, a critical gap remains in systematically linking chemotype-specific biosynthetic pathways—particularly in light of recent genomic and transcriptomic insights—with a structured evaluation of the evidence for their health benefits. This review addresses this gap by integrating current knowledge on chemical composition, biosynthetic pathways, and pharmacological effects. It provides a comprehensive analysis of how chemotypic variation, driven by genetic and epigenetic factors, correlates with demonstrated bioactivities, thereby offering a more nuanced and evidence-based perspective on their therapeutic potential. Furthermore, this review identifies existing research challenges and proposes future directions to fully realize the capacity of *Mentha* EOs in promoting human health and well-being across various industries.

## 2. Literature Search Strategy

To ensure a comprehensive and unbiased coverage of the current knowledge on *Mentha* EOs, a systematic literature search was conducted. The methodology was designed to identify, select, and critically appraise relevant scientific publications pertaining to the chemotypic diversity, biosynthetic pathways, and health-promoting activities of *Mentha* EOs.

### 2.1. Search Strategy and Data Sources

An extensive electronic literature search was performed across three major scholarly databases: Web of Science Core Collection, Scopus, and PubMed/Medline. The search was limited to articles published in English between January 2018 and July 2024 to focus on the most recent advances in the field. The search strategy utilized a combination of keywords and Boolean operators to maximize relevance and coverage. The core search string was constructed as follows:

(“*Mentha*” OR “mint” OR “peppermint” OR “spearmint” OR “pennyroyal”) AND (“essential oil” OR “volatile oil” OR “volatile compound”) AND (“chemotype” OR “chemical composition” OR “biosynthesis” OR “MEP pathway” OR “terpene synthase” OR “health” OR “bioactive” OR “antimicrobial” OR “antioxidant” OR “anti-inflammatory *” OR “anticancer” OR “digestive” OR “cognitive”)

This query was adapted to the specific syntax of each database. The reference lists of key review articles and primary research papers were also manually screened to identify additional relevant publications that may not have been captured by the electronic search.

### 2.2. Study Selection and Inclusion Criteria

The retrieved records were screened in a two-stage process based on their titles/abstracts and subsequently by full-text assessment. Studies were included if they met the following pre-defined criteria: (1) Population/Subject: Studies focused on plants of the genus *Mentha* and their derived EOs; (2) Intervention/Exposure: Investigations into the chemical composition, chemotypic variation, biosynthetic pathways (e.g., MEP pathway, enzymatic regulation), or biological/pharmacological activities of *Mentha* Eos; (3) Outcomes: Reported quantitative data on EO composition (e.g., GC-MS analysis), mechanistic insights into biosynthesis (e.g., gene expression, enzyme activity), or results from in vitro and in vivo assays demonstrating health-promoting effects; (4) Study Type: Original research articles, review articles, and meta-analyses published in peer-reviewed journals.

### 2.3. Exclusion Criteria

Studies were excluded based on the following criteria: (1) Publications not in the English language; (2) Studies where the full text was not accessible; (3) Articles focusing on non-essential oil extracts (e.g., aqueous or ethanolic extracts) without direct relevance to volatile compounds, unless for comparative purposes in biological activity sections; (4) Conference abstracts, patents, or unpublished theses to ensure the quality and verifiability of the data synthesized.

### 2.4. Data Extraction and Synthesis

From the included studies, relevant data were extracted into a standardized form, including: author(s) and publication year, *Mentha* species studied, key chemical constituents and their percentages, methodologies used for analysis (e.g., GC-MS), key findings related to biosynthesis, and details of biological activities (e.g., assay type, results, mechanisms of action). The data were then synthesized narratively to present a coherent overview of the current state of knowledge, identify consensus and controversies in the literature, and highlight gaps for future research, as presented in the subsequent sections of this review.

The final selection of literature provides a robust foundation for critically evaluating the chemotype-dependent biosynthesis and the evidence supporting the diverse health-promoting activities of *Mentha* EOs.

## 3. Chemical Composition of *Mentha* EOs

The therapeutic and aromatic properties of *Mentha* EOs are directly attributable to their complex chemical composition, which is predominantly characterized by a rich array of monoterpenes and their oxygenated derivatives. These volatile compounds are synthesized and stored in specialized glandular trichomes on the plant surface, and their specific profiles define the distinct chemotypes observed across different *Mentha* species and even within the same species under varying conditions [17,33]. Understanding this chemical diversity, the factors influencing it, and the analytical methods employed for its characterization is fundamental to appreciating the multifaceted utility of *Mentha* EOs.

### 3.1. Structural Diversity of Major Monoterpenes

**Figure 2:**
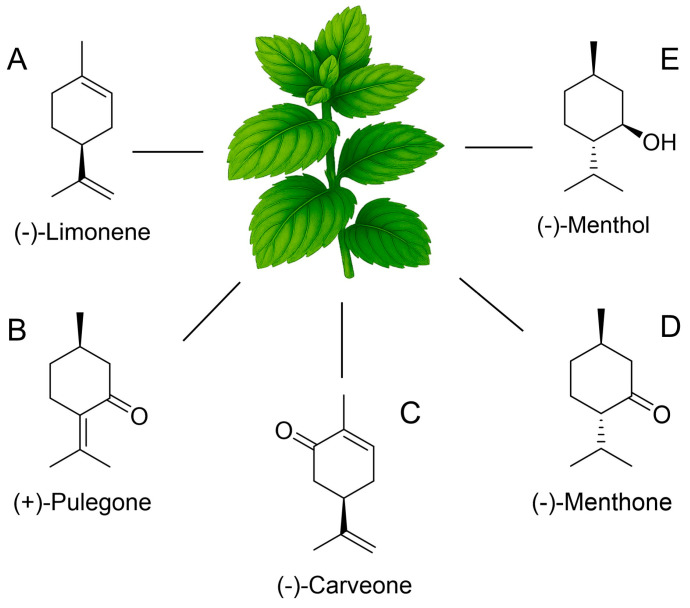
Chemical Structures of Major Monoterpenes in *Mentha* EOs.

The EOs of *Mentha* species are renowned for their high content of monoterpenes, which are C10 compounds derived from isoprene units. These compounds exhibit significant structural diversity, leading to a wide range of aromas and biological activities. Among the most prominent monoterpenes found in *Mentha* EOs are menthol, menthone, pulegone, carvone, and limonene, each contributing to the unique sensory and therapeutic profile of specific mint varieties (Figure 2). For instance, *Mentha piperita* (peppermint) EO is consistently characterized by a high concentration of menthol (Figure 2E) and menthone (Figure 2D), often constituting around 50% of its total composition [11,34,35]. This menthol-rich profile is responsible for the characteristic cooling sensation and strong, refreshing aroma associated with peppermint, making it highly valued in food, cosmetic, and pharmaceutical industries. Similarly, the EO of *Mentha arvensis* is also dominated by menthol (typically over 60%), along with menthone (4–18%), underscoring the significance of this monoterpene in defining specific mint chemotypes [11,36]. The presence of menthol, often alongside menthone, is a defining feature that distinguishes these species and dictates their primary applications [15].

In contrast, *Mentha spicata* (spearmint) EO presents a distinct chemical signature, with carvone (Figure 2C) typically being the major component, often exceeding 60% of the oil, accompanied by limonene (Figure 2A) [11,13,23,32,34,37]. This carvone-rich profile imparts a sweeter, milder, and less pungent aroma compared to peppermint, making spearmint EOs particularly popular in chewing gums, toothpastes, and certain culinary applications. The study by Piras et al. (2019) [13] specifically identified carvone at 62.9% in *M. spicata* EO from Sardinia, underscoring its dominance. Similarly, Oliveira Filho et al. (2023) [37] reported carvone at 68.88% and limonene at 20.34% in *M. spicata* EO, further exemplifying this chemotype. The presence of limonene, a cyclic monoterpene hydrocarbon, often accompanies carvone and contributes to the overall citrusy undertones of spearmint.

Another significant chemotype is represented by *Mentha pulegium* (pennyroyal), where pulegone (Figure 2B) is the overwhelmingly dominant compound, frequently accounting for over 70% of the EO composition [13,38,39,40,41,42]. For instance, El abdali et al. (2024) [38] reported pulegone at 72.05% in Moroccan *M. pulegium* EO, while Piras et al. (2019) [13] found it at 86.2% in Sardinian samples. Messaoudi et al. (2021) [39] also identified pulegone as the major component at 74.81% in *M. pulegium* from Algeria. This high concentration of pulegone gives pennyroyal a strong, somewhat camphoraceous aroma, but also raises concerns regarding its toxicity due to pulegone’s hepatotoxic potential, which necessitates careful consideration for its applications. Other oxygenated monoterpenes like menthone and piperitone can also be present in notable amounts in *M. pulegium* EOs, further shaping its profile [39].

Limonene, a cyclic monoterpene hydrocarbon, is a common constituent in many citrus EOs but also appears in varying amounts in *Mentha* species. While not typically the dominant compound in most *Mentha* EOs, its presence can influence the overall aroma profile, adding a fresh, citrusy note. Sarri et al. (2024) highlighted limonene as one of the terpenes demonstrating strong nematicidal potential, underscoring its broad biological relevance beyond just aroma [43].

**Figure 3:**
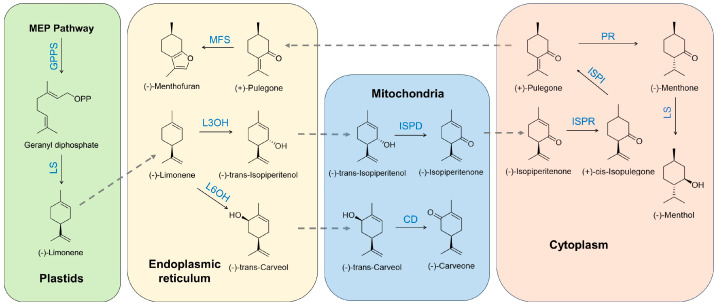
Biosynthetic Pathway of Key Monoterpenoids in *Mentha* Species. The enzymatic reaction steps are catalyzed by geranyl-diphosphate synthase (GPPS), (−)-limonene synthase (LS), (−)-limonene 6-hydroxylase (L6OH), (−)-trans-carveol dehydrogenase (CD), (−)-limonene 3-hydroxylase (L3OH), isopiperitenol dehydrogenase (ISPD), (−)-isopiperitenone reductase (ISPR), (+)-cis-isopulegone isomerase (ISPI), (+)-menthofuran synthase (MFS), (−)-pulegone reductase (PR), and (−)-menthol dehydrogenase (MD).

*Mentha suaveolens* (apple mint) presents yet another distinct chemotype, often characterized by the prevalence of piperitenone (Figure S2A) and piperitenone oxide (Figure S2B), rather than menthol or carvone [17,44,45]. Yang et al. (2024) [17] highlighted that *M. suaveolens* primarily produces piperitenone oxide, with low transcription levels of isopiperitenone reductase (ISPR) (Figure 3), an enzyme crucial for menthol biosynthesis. Bouyahya et al. (2019) [44] reported piperitenone oxide (56.28%), piperitenone (11.64%), and pulegone (6.16%) as major components in *M. suaveolens* EOs. This demonstrates how subtle shifts in biosynthetic pathways can lead to entirely different dominant compounds and, consequently, different chemotypes and applications. *Mentha longifolia* also exhibits variability, with pulegone, 1,8-cineole (Figure S2C), and L-menthone identified as major compounds, though their proportions can vary seasonally [28]. Brahmi et al. (2024) [46] identified p-Menth-4(8)-en-3-one (50.48%) (Figure S2D) as predominant in *Mentha subtomentella*, showcasing further diversity within the genus. The presence of 1,8-cineole, also known as eucalyptol, is notable in some *Mentha* species, contributing a camphoraceous, fresh aroma, as seen in *M. spicata* and *M. piperita* to a lesser extent [31,32]. This rich structural diversity of monoterpenes and their oxygenated derivatives underscores the complexity and versatility of *Mentha* EOs, with each chemotype offering unique properties for various applications.

### 3.2. Chemotypic Variations and Influencing Factors

The chemical composition of *Mentha* EOs is not static but is subject to considerable variability, leading to the existence of distinct chemotypes. These chemotypes are defined by the quantitative and qualitative differences in their major constituents, which in turn dictate their aroma, biological activity, and commercial value [2,47]. This variability is influenced by a confluence of factors, including genetic background, geographical origin, environmental conditions, developmental stage of the plant, and even the specific extraction method employed [14,16,28,48].

Genetic factors play a foundational role in establishing the potential chemotype of a *Mentha* plant. Different *Mentha* species inherently produce distinct major monoterpenes due to their unique genetic endowments, which encode the specific enzymes involved in terpenoid biosynthesis. For instance, *M. piperita* is genetically predisposed to produce menthol and menthone, while *M. spicata* is geared towards carvone and limonene production [49,50]. However, even within a single species, genetic variations among different accessions can lead to diverse chemotypes [51]. The study by Yang et al. (2024) [17] on *M. suaveolens* ‘Variegata’ highlights the significance of genetic variations, specifically structural variations and low transcription levels of key enzymes like ISPR, in dictating the accumulation of specific monoterpenoids such as piperitenone oxide over menthol. This research, which provided a haplotype-resolved gap-free genome assembly, offers novel insights into the molecular basis of monoterpenoid diversification, demonstrating how genetic predispositions can lead to distinct chemotypes. Similarly, Mustafa et al. (2024) [16] investigated 20 accessions of *M. longifolia* from different regions of Iran and Iraq, finding significant differences in EO content and constituents. Their cluster analysis, which did not strictly align with geographical distance, suggested a more prominent role of genetic factors compared to habitat conditions in differentiating these accessions, further emphasizing the genetic underpinning of chemotypic variation [52].

Environmental conditions are another critical determinant of EO composition. Factors such as climate, soil composition, altitude, and water availability can significantly modulate the expression of biosynthetic genes and the activity of enzymes, thereby altering the final chemical profile. Haddou et al. (2023) [14] investigated the impact of irrigation water quality on the secondary metabolites and chemical profile of *Mentha* × *piperita* L. essential oil. Their study revealed notable variations in the essential oil composition of plants subjected to different irrigation sources. Specifically, essential oil EO1, derived from plants irrigated with treated wastewater, contained a total of twelve distinct compounds. This oil was characterized by carvone as its primary component, accounting for 51.35% of the total composition, followed by D-limonene (17.88%), eucalyptol (10.69%), β-caryophyllene (4.39%), and Germacrene D (5.21%). In contrast, essential oil EO2, extracted from plants nurtured with well water, exhibited a more diverse chemical profile comprising eighteen identified compounds. Carvone also predominated in EO2, but at a significantly higher concentration of 76.89%, while other major constituents included eucalyptol (5.22%), D-limonene (3.27%), β-caryophyllene (3.02%), and Germacrene D (2.97%). Finally, essential oil EO3, obtained from plants irrigated with river water, contained seventeen compounds, with carvone again being the dominant component at 77.17%, followed by 1,8-epoxy-p-menthane (5.06%), D-limonene (3.42%), α-cubebene (2.98%), and β-caryophyllene (2.93%). Collectively, these results demonstrate that even subtle differences in water quality, as a form of environmental stressor or nutritional influence, can significantly alter the production of specific monoterpenes and sesquiterpenes in peppermint. This finding holds crucial implications for cultivation practices, as it suggests that controlled irrigation strategies can be employed as a deliberate tool to enhance the yield of desired metabolites. Seasonal variations also contribute to chemotypic shifts. Zouari-Bouassida et al. (2018) [28] observed seasonal variation in the EO composition of *M. longifolia* leaves grown in Tunisia, noting that the highest EO yield was recorded in spring, and the major compounds like pulegone, 1,8-cineole, and L-menthone varied in proportion depending on the season. This highlights the dynamic nature of EO production in response to environmental cues throughout the plant’s life cycle.

Geographical origin, often intertwined with local environmental conditions and genetic diversity, also plays a significant role. Different regions may harbor distinct ecotypes or landraces of *Mentha* species that have adapted to local conditions, leading to unique chemotypes. Afkar and Somaghian (2024) [30] analyzed three different ecotypes of *M. longifolia* collected from Lorestan province, Iran, and found variations in EO concentration and specific biological activities, although the overall sensitivity of tested bacteria to the EOs was similar across ecotypes. This suggests that while the overall biological activity might be conserved, the specific chemical nuances can differ based on geographical provenance. The study by El abdali et al. (2024) [38] on Moroccan *M. pulegium* identified pulegone as the major component, a finding consistent with other *M. pulegium* studies from different regions, but the precise percentages and minor components can still exhibit regional specificities.

Finally, the method of EO extraction can significantly influence the perceived chemical composition and yield, although this relates more to analytical representation than the plant’s inherent biosynthetic capacity. Different extraction techniques, such as hydrodistillation (HD), steam distillation (SD), and microwave-assisted distillation (MAD), vary in their efficiency at extracting specific compounds and can potentially cause degradation of thermolabile constituents due to differences in temperature and processing time [39,53]. A comparative study by Messaoudi et al. (2021) [39] on *M. pulegium* demonstrated these methodological impacts clearly. While pulegone remained the dominant component across all three extraction methods (HD, SD, and MAD), its percentage varied (74.81%, 73.17%, and 71.52%, respectively), as did the proportions of other key compounds like menthone and piperitone. Furthermore, the total identified oil content was highest with HD (97.73%), followed by SD (92.80%) and MAD (78.95%), and the extraction yield also followed this pattern (0.204%, 0.200%, and 0.175%, respectively). MAD, however, offered a significant advantage in extraction time (30 min versus 3 h for HD/SD). These findings underscore the critical importance of consistent and well-documented extraction methodologies in both research and industrial production to ensure reproducible chemical profiles, accurate chemotyping, and reliable biological activities.

### 3.3. Analytical Methodologies for EO Characterization

Accurate and comprehensive characterization of *Mentha* EOs is indispensable for understanding their chemotypic variations, correlating chemical profiles with biological activities, and ensuring quality control for commercial applications. The complexity of EOs, which are mixtures of dozens to hundreds of volatile compounds, necessitates sophisticated analytical techniques capable of both separation and identification. Gas Chromatography-Mass Spectrometry (GC-MS) stands as the gold standard for this purpose, providing unparalleled detail in the qualitative and quantitative analysis of EO constituents [14,15,25,31,32,34,37,38,39,40,41,44,45,50,52,54,55].

Gas Chromatography–Mass Spectrometry (GC-MS) integrates the powerful separation capability of gas chromatography (GC) with the high-specificity detection of mass spectrometry (MS). In this technique, the EO sample is vaporized and transported through a chromatographic column using an inert carrier gas. Component separation is achieved based on differential partitioning between the mobile and stationary phases. As individual compounds elute from the column, they enter the mass spectrometer, where they undergo ionization and fragmentation into characteristic mass spectra. These fragmentation patterns serve as molecular fingerprints, which are compared against established spectral libraries to enable confident identification of individual compounds. Additionally, the relative abundance of each constituent can be estimated based on chromatographic peak areas.

GC-MS has been extensively applied in the chemical profiling of *Mentha* EOs. For example, El abdali et al. (2024) [38] identified thirteen components in *M. pulegium* EO, representing 97.74% of the total composition. Pulegone (72.05%) was the predominant compound, followed by 8-hydroxy-p-menthan-3-one (5.97%), imidazolidine (3.23%), and piperitenone (3.02%). Monoterpenes constituted the most abundant chemical class, accounting for 83.85% of the oil. In a comparative study, Wu et al. (2019) [34] analyzed the EOs of *M. piperita*, *M. spicata*, and *M. gracilis* using GC-MS, revealing distinct chemotypic variations. *M. piperita* EO was characterized by high concentrations of menthol (38.45%) and menthone (21.80%), with 1,8-cineole (5.62%) and neo-menthol (4.19%) as minor constituents. In contrast, both *M. spicata* and *M. gracilis* EOs were rich in carvone (70.36% and 70.91%, respectively) and limonene (6.96% and 13.96%, respectively). These results are consistent with the findings of Oliveira Filho et al. (2023) [37], who also identified carvone (68.88%) and limonene (20.34%) as the major components in *M. spicata* EO.

Similarly, Al-Mijalli et al. (2022) [45] used GC-MS to characterize *M. suaveolens* EO, reporting pulegone (37.16%) and pyrazines (33.81%) as the primary constituents, along with limonene (11.19%) and umbellulone (6.09%). Messaoudi et al. (2021) [39] further identified 26 compounds in *M. pulegium* EO, with pulegone (74.81%) as the dominant component, followed by menthone (13.01%), piperitone (3.82%), limonene (1.55%), and *cis*-isopulegone (1.31%); remaining components each accounted for less than 1%.

Collectively, these studies underscore the indispensable role of GC-MS in elucidating the chemical diversity of mint EOs. The technique provides high sensitivity, excellent selectivity, and the ability to identify a broad spectrum of volatile and semi-volatile compounds, making it particularly suitable for detailed chemotypic analysis.

Beyond identification and quantification, these analytical techniques are crucial for assessing the impact of various factors on EO composition. For instance, the effect of different drying methods on *Mentha × piperita* EO was evaluated using GC/MS, revealing significant variations in menthol, menthone, and menthofuran concentrations [15]. Similarly, the chemical composition of *M. piperita* EO irrigated with different water sources was determined by GC/MS, showing notable variations among the EOs [14].

In the chemical characterization of EOs, Gas Chromatography with Flame Ionization Detection (GC-FID) is frequently employed in conjunction with GC-MS, particularly for quantitative analysis. While GC-MS excels in identifying compound structures, GC-FID provides highly sensitive and linear detection for most organic compounds, making it especially suitable for the precise quantification of constituents previously identified by GC-MS. This complementary approach has been effectively applied in the analysis of *Mentha* EOs. For instance, Messaoudi et al. (2021) [39] and Cheraif et al. (2020) [40] both utilized GC-FID alongside GC-MS to analyze *M. pulegium* EOs. Their results consistently demonstrated that the EO is overwhelmingly dominated by oxygenated monoterpenes (exceeding 90% of the total composition) and exhibits a distinct pulegone chemotype, with pulegone itself being the most abundant compound (76.9%) [56]. This confirms the utility of GC-FID in obtaining accurate quantitative data on identified constituents.

Similarly, Biltekin et al. (2023) [32] employed integrated GC-FID and GC-MS analysis to characterize *M. spicata* EO, identifying carvone (72.8%), limonene (12.6%), 1,8-cineole (2.2%), myrcene (1.3%), and *trans*-dihydrocarvone (1.0%) as its major components. The analysis of seasonal variations in *M. longifolia* EOs also relied on GC-MS and GC-FID to track changes in major compounds such as pulegone and piperitenone oxide throughout the vegetative cycle [57]. Together, these studies underscore the value of combining both techniques for comprehensive and reliable chemical profiling of EOs.

While GC-MS and GC-FID offer detailed compositional analysis, there is a growing need for rapid, cost-effective, and non-destructive methods for routine quality control and screening, especially in industrial settings. Attenuated Total Reflectance–Fourier Transform Infrared (ATR-FTIR) spectroscopy coupled with multivariate analyses has emerged as a promising alternative for such applications. This technique measures the absorption of infrared light by the sample, providing a unique spectral fingerprint that reflects the molecular vibrations of its constituents. By applying chemometric models, it is possible to correlate specific spectral features with the presence and concentration of key compounds or even to distinguish between different EOs or detect adulteration. Rodríguez et al. (2018) [58] successfully developed a rapid ATR-FTIR method for quantifying clove and spearmint (*M. spicata*) EOs encapsulated in a complex organic matrix. They demonstrated that despite the matrix complexity, this method, coupled with univariate or multivariate regression models, could accurately quantify the EOs. For spearmint EO, specific bands (799, 885, and 1680–1676 cm^−1^) were identified as suitable for prediction, with high accuracy. The study highlighted that ATR-FTIR not only saved time compared to GC-FID but also offered the capability to monitor the EO profile, making it suitable for routine quality control. Similarly, Taylan et al. (2021) [59] utilized ATR-FTIR spectroscopy coupled with chemometrics (HCA, PCA, PLSR, PCR) for rapid screening of *M. spicata* EO and L-menthol adulteration in *M. piperita* EOs. They successfully distinguished authentic *M. piperita* from adulterated samples and accurately calculated adulterant contents, demonstrating the method’s robustness, reliability, and economic advantages for monitoring adulteration, a critical issue in the high-value EO market. The comparative advantage of ATR-FTIR lies in its speed, minimal sample preparation, and non-destructive nature, making it highly suitable for rapid screening and quality assurance, although it may not provide the same level of detailed compound identification as GC-MS. The integration of these advanced analytical methodologies, from highly detailed chromatographic techniques to rapid spectroscopic screening, provides a comprehensive toolkit for unraveling the chemical complexity of *Mentha* EOs and ensuring their quality and efficacy for diverse applications.

## 4. Biosynthetic Pathways of Major Monoterpenes

The remarkable diversity of monoterpenes found in *Mentha* EOs is a testament to the sophisticated biosynthetic machinery within the plant. These volatile compounds are secondary metabolites, meaning they are not directly involved in the primary growth and development of the plant but play crucial roles in ecological interactions, such as defense against herbivores and pathogens, and attraction of pollinators [60,61]. The biosynthesis of monoterpenes in *Mentha* species primarily occurs via the plastidial methylerythritol phosphate (MEP) pathway, a complex cascade of enzymatic reactions that are tightly regulated at genetic and epigenetic levels (Figure 3) [21,56]. Understanding these pathways is not only fundamental to plant biochemistry but also offers promising avenues for metabolic engineering to optimize the production of desired chemotypes [62].

### 4.1. Overview of the Plastidial MEP Pathway

Monoterpenes, being C10 compounds, are derived from two C5 isoprene units. In plants, these isoprene units are primarily supplied by two distinct pathways: the mevalonate (MVA) pathway, which operates in the cytosol and is responsible for sesquiterpene and triterpene biosynthesis, and the MEP pathway, which is localized in the plastids and is the main route for monoterpene and diterpene production [60]. For *Mentha* EOs, the MEP pathway is of paramount importance as it provides the direct precursors for the characteristic monoterpenes like menthol, menthone, pulegone, carvone, and limonene.

The MEP pathway (Figure S1) begins with pyruvate and glyceraldehyde-3-phosphate, which are primary metabolites derived from photosynthesis. Through a series of enzymatic steps, these precursors are converted into the universal C5 isoprenoid building blocks: isopentenyl pyrophosphate (IPP) and its isomer, dimethylallyl pyrophosphate (DMAPP). These two molecules are the fundamental units from which all terpenoids are constructed. The first committed step in monoterpene biosynthesis involves the head-to-tail condensation of one molecule of IPP and one molecule of DMAPP, catalyzed by an enzyme called geranyl pyrophosphate synthase (GPPS). This reaction yields geranyl pyrophosphate (GPP), a C10 precursor that serves as the immediate substrate for all monoterpenes [63].

The subsequent steps in the pathway involve the cyclization and modification of GPP by a diverse family of enzymes, primarily terpene synthases (TPSs) [20] and cytochrome P450 monooxygenases (CYP450s). These enzymes are responsible for the vast structural diversity observed in monoterpenes. For instance, GPP can be cyclized to form various cyclic monoterpene hydrocarbons, such as limonene, which is a key intermediate in the biosynthesis of many oxygenated monoterpenes in *Mentha* species. Limonene can then undergo further enzymatic modifications, including hydroxylation and dehydrogenation, to yield compounds like carvone, pulegone, menthone, and menthol. The specific sequence and activity of these modifying enzymes dictate the final chemotype of the plant. The study by Qi et al. (2018) [18] on *Mentha canadensis* L. and Huang et al. (2025) [19] on *M. arvensis* L. both identified “Monoterpenoid biosynthesis” as a significantly enriched pathway in their transcriptome analyses, underscoring the central role of this pathway in *Mentha* species. These studies also identified key genes involved in monoterpene biosynthesis, such as GPPS, LS (limonene synthase), L3OH (limonene-3-hydroxylase), and ISPR, which are direct participants in the conversion of GPP to various oxygenated monoterpenes (Figure 3). The overall efficiency and specific branches taken within the MEP pathway are critical determinants of the quantity and quality of EOs produced by *Mentha* plants.

### 4.2. Enzymatic Regulation of Terpenoid Biosynthesis

The enzymatic machinery is the primary driver of terpenoid biosynthesis, dictating not only the rate of production but also the specific chemical structures of the resulting compounds. The diversity of *Mentha* chemotypes, characterized by the predominance of compounds like menthol, carvone, or pulegone, is a direct consequence of the differential expression and activity of key enzymes in the MEP pathway. TPSs are central to this process, catalyzing the cyclization of GPP into various monoterpene hydrocarbons [64,65]. For example, LS converts GPP into limonene, a pivotal intermediate in many *Mentha* chemotypes (Figure 3). The presence and activity of specific TPS isoforms can determine whether a plant primarily produces cyclic or acyclic monoterpenes, or which specific cyclic structure is formed.

Following the initial cyclization, a suite of modifying enzymes, predominantly cytoCYP450s and reductases, further transform these hydrocarbon precursors into the oxygenated monoterpenes that are characteristic of *Mentha* EOs [21]. These enzymes introduce hydroxyl groups, carbonyl groups, and double bonds, leading to the formation of compounds like menthol, menthone, pulegone, and carvone (Figure 3) [66,67]. The sequential action of these enzymes is highly specific and often rate-limiting, meaning that the activity of a single enzyme can profoundly impact the final chemical profile. A compelling example of this enzymatic regulation is highlighted in the study by Yang et al. (2024) [17] on *M. suaveolens* ‘Variegata’. This species primarily accumulates piperitenone oxide rather than menthol. The researchers identified three genes encoding ISPR, a key rate-limiting enzyme in menthol biosynthesis. Crucially, they found that the transcription levels of ISPR were low in *M. suaveolens*, despite other terpenoid biosynthesis genes being expressed. This low ISPR activity directly accounts for the accumulation of piperitenone oxide, as the pathway to menthol is effectively bottlenecked at this step. This finding provides a clear illustration of how the activity of a specific enzyme can define the chemotype of a *Mentha* species.

Similarly, the biosynthesis of carvone, the major component in *M. spicata*, involves a different set of enzymes that convert limonene into carvone via intermediates like limonene-6-hydroxylase and carvone reductase (Figure 3). Conversely, the pulegone-rich chemotype of *M. pulegium* is governed by enzymes that channel GPP towards pulegone synthesis, potentially involving different limonene hydroxylases and subsequent dehydrogenases. The balance and specificity of these enzymatic reactions are critical. For instance, if an enzyme responsible for converting pulegone to menthone or menthol is less active or absent, pulegone will accumulate. Conversely, if the pathway proceeds efficiently through these steps, menthol will be the dominant end-product. The identification of specific enzymes and their functional characterization, as exemplified by the ISPR in *M. suaveolens* [17], is crucial for understanding the molecular basis of chemotypic diversity and for potential biotechnological applications aimed at manipulating EO composition. The intricate interplay of these enzymes, their substrate specificities, and their catalytic efficiencies collectively orchestrate the complex chemical profiles observed in *Mentha* EOs.

### 4.3. Genetic and Epigenetic Regulation of Biosynthetic Genes

The production of monoterpenes in *Mentha* species is not solely dependent on the presence of biosynthetic enzymes but is also intricately controlled by genetic and epigenetic regulatory mechanisms that govern the expression of the genes encoding these enzymes. This multi-layered regulation allows plants to fine-tune their EO composition in response to developmental cues and environmental stimuli, contributing significantly to chemotypic variations.

At the genetic level, the organization and sequence of genes encoding key biosynthetic enzymes are fundamental. The availability of high-quality genome assemblies is revolutionizing our understanding of these genetic underpinnings. Yang et al. (2024) [17] achieved a significant breakthrough by providing a haplotype-resolved gap-free genome assembly for *M. suaveolens* ‘Variegata’. This detailed genomic resource allowed for the detection of numerous structural variations and, crucially, demonstrated that genes involved in terpenoid biosynthesis were affected by these variations. Such genomic insights are vital for identifying the genetic loci responsible for chemotypic differences and for understanding how gene duplication, deletion, or rearrangement can lead to novel biosynthetic capabilities or altered expression patterns. The identification of specific genes encoding ISPR and the observation of their low transcription levels in *M. suaveolens* directly link genetic regulation to the accumulation of piperitenone oxide, providing a clear example of how gene expression dictates the final EO profile [17]. This type of genomic information is invaluable for molecular marker-assisted breeding, enabling the selection of *Mentha* varieties with desired chemotypes.

Transcriptional regulation, mediated by transcription factor (TF), plays a pivotal role in controlling the expression of biosynthetic genes. Phytohormones, such as jasmonates and auxins, are key signaling molecules that modulate these transcriptional networks. Methyl jasmonate (MeJA), a derivative of jasmonic acid (JA), is a well-known elicitor of secondary metabolite biosynthesis in many plants, including *Mentha* species. Qi et al. (2018) [18] conducted a transcriptome analysis of *M*. *canadensis* L. in response to MeJA treatment, identifying 4430 differentially expressed genes (DEGs). Their Kyoto Encyclopedia of Genes and Genomes (KEGG) enrichment analysis highlighted “Monoterpenoid biosynthesis” as one of the most significantly enriched pathways, indicating a strong transcriptional response to MeJA. They further analyzed DEGs involved in JA signal transduction, transcription factors, and monoterpene biosynthesis, identifying 9 orthologous genes involved in menthol biosynthesis. This study provided crucial insights into the transcriptional regulation of menthol biosynthesis in *M. canadensis*.

Building upon this, Huang et al. (2025) [19] investigated the regulatory mechanism of MeJA-induced monoterpenoid biosynthesis in *M. arvensis* L. They found that exogenous application of MeJA significantly increased EO content, yield, and peltate glandular trichome (PGT) density in a dose-dependent manner. PGTs are the specialized structures where EOs are synthesized and stored, so an increase in their density directly correlates with higher EO production. Transcriptome analysis revealed 4659 DEGs, with “Monoterpenoid biosynthesis” again being a highly enriched pathway. Crucially, key genes involved in JA signaling (JAZs and MYCs) and monoterpenoid biosynthesis (GPPSs, LSs, L3OHs, and ISPRs) were significantly up-regulated. Co-expression analysis and weighted gene co-expression network analysis (WGCNA) identified transcription factors such as AP2/ERF, WRKY, MYB, and bHLH as crucial regulators of MJ-mediated monoterpenoid biosynthesis. These findings provide a detailed molecular understanding of how MeJA signaling pathways activate specific TFs, which in turn upregulate the expression of genes encoding biosynthetic enzymes, leading to increased monoterpenoid accumulation.

Beyond jasmonates, other phytohormones also exert regulatory control. Reddy et al. (2024) [33] identified a non-canonical Aux/IAA gene, MsIAA32, in *M. spicata* (spearmint) that is involved in the development of PGT. Auxin signaling, typically involving canonical Aux/IAA proteins, is known to control various aspects of plant growth. However, MsIAA32, lacking the TIR1-binding domain, represents a novel regulatory mechanism. Its interaction with canonical Aux/IAAs (MsIAA3, MsIAA4) and an ARF (MsARF3) suggests a complex auxin-mediated signaling pathway. Altered expression of R2R3-MYB gene MsMYB36 and cyclin gene MsCycB2-4 in MsIAA32-suppressed plants indicates these as possible downstream targets. This research establishes a role for non-canonical Aux/IAA-mediated auxin signaling in PGT development, thereby indirectly influencing EO production by regulating the very structures that synthesize and store these compounds.

Epigenetic mechanisms, chiefly mediated through DNA methylation and histone modifications, represent a crucial layer of gene regulation that modulates transcriptional activity without changing the primary DNA sequence. These mechanisms directly influence chromatin architecture and its accessibility to DNA-binding proteins, including transcription factors (TFs), thereby fine-tuning gene expression. DNA methylation entails the covalent addition of a methyl group to cytosine residues, whereas histone modifications encompass a range of post-translational chemical alterations—such as acetylation, methylation, phosphorylation, and sumoylation—targeting the N-terminal tails of histone proteins. Notably, methylation at specific lysine (K) residues on histones H3 and H4 can produce distinct and occasionally antagonistic effects on transcriptional output [68]. In addition to these structural variations, the regulation of gene expression through various non-coding RNAs (ncRNAs) is also an epigenetic mechanism of gene regulation. Although the specific involvement of epigenetic regulation in Mentha monoterpene biosynthesis has not been fully elucidated in the existing literature, insights from plant secondary metabolism suggest that such mechanisms may contribute to stable chemotypic differentiation or long-term adaptive responses to environmental stimuli. The complex interplay between genetic background, transcriptional control mediated by TFs and phytohormones, and potential epigenetic modifications forms a highly dynamic and plastic regulatory network that orchestrates monoterpene biosynthesis in *Mentha* species. This integrated framework ultimately underlies the emergence and persistence of distinct chemotypes. A deeper understanding of these multi-layered regulatory processes will be essential for advancing metabolic engineering approaches and developing precision breeding strategies for *Mentha* cultivars.

## 5. Health-Beneficial Effects of *Mentha* EOs

*Mentha* EOs have garnered significant attention in scientific research due to their extensive array of health-promoting properties, which underpin their traditional uses and highlight their potential for modern therapeutic applications. The diverse chemical profiles, particularly the varying proportions of key monoterpenes across different *Mentha* chemotypes, contribute to a broad spectrum of biological activities, including antimicrobial, antioxidant, anti-inflammatory, neurological, digestive, respiratory, cognitive-enhancing, and even emerging anticancer effects (Table 1). This section critically evaluates the evidence supporting these multifaceted health benefits.

### 5.1. Antimicrobial and Antifungal Properties

The antimicrobial and antifungal properties of *Mentha* EOs (EOs) are among their most well-documented biological activities, positioning them as promising natural alternatives to synthetic agents in medicine. Their efficacy is primarily attributed to major monoterpenoid constituents—such as menthol, carvone, pulegone, and limonene—which disrupt microbial cell membranes and inhibit key cellular processes [75,76,77].

Numerous studies have demonstrated the broad-spectrum antibacterial activity of *Mentha* EOs against human pathogens. *M. piperita* (peppermint) EO, rich in menthol, has shown significant effects against oral and systemic pathogens. Karadağ et al. (2024) [35] reported that *M. piperita* EO enhanced the efficacy of chlorhexidine against *Streptococcus mutans* and *Staphylococcus aureus* when combined with *Pelargonium graveolens* EO, indicating potential synergies with conventional antimicrobials in oral care. Separately, Gebauer et al. (2024) [11] confirmed the moderate toxicity of *M. piperita* EO against *S. aureus* (ATCC 25923), supporting its role in topical or systemic antibacterial strategies.

*M. spicata* (spearmint) EO, characterized by high carvone content, also exhibits notable antimicrobial properties. Landeo-Villanueva et al. (2023) [23] demonstrated that *M. spicata* EO significantly inhibited *Streptococcus mutans* planktonic growth (MIC = 1.8484 mg/mL) and reduced biofilm biomass under conditions mimicking dental plaque, underscoring its potential in preventing dental caries. In the same study, the oil showed inhibition zones of 18.3 ± 0.47 mm in agar diffusion assays.

*M. pulegium* (pennyroyal) EO, dominated by pulegone, has displayed consistent antibacterial and antifungal effects against clinically relevant strains. El abdali et al. (2024) [38] reported potent activity of Moroccan *M. pulegium* EO against *S. aureus* and *E. coli*, with MIC values of 3.058 mg/mL and 6.076 mg/mL, respectively. The same study also confirmed its antifungal efficacy against *Candida albicans* (MIC = MBC = 3.063 mg/mL). These findings are corroborated by Messaoudi et al. (2021) [39], who documented significant antimicrobial and anti-*Candida* activity for *M. pulegium* EO.

Additional species such as *M. suaveolens* and *M. longifolia* have also shown medically relevant antimicrobial profiles. Bouyahya et al. (2019) [44] found that *M. suaveolens* EO, rich in piperitenone oxide, exhibited antibacterial action against *Staphylococcus aureus* and *Listeria monocytogenes*, with particular sensitivity observed in *S. aureus*. Al-Mijalli et al. (2022) [45] further reported that *M. suaveolens* EO was effective against a majority of tested clinical bacterial isolates.

In terms of antifungal applications, several *Mentha* EOs have shown promise against human fungal pathogens. Piras et al. (2019) [13] demonstrated that *M. spicata* and *M. pulegium* EOs strongly inhibited *Candida albicans* germ tube formation at sub-MIC levels (0.16 μL/mL), indicating an ability to suppress virulence factors. *M. spicata* EO was particularly effective against dermatophytes, including *Trichophyton rubrum* and *T. verrucosum* (MIC = 0.32 μL/mL). Brahmi et al. (2024) [46] also reported notable antifungal activity for *M. subtomentella* EO, though its primary activity was against non-human fungal strains.

In summary, the collective evidence supports the potential of *Mentha* EOs as valuable natural agents for medical applications—particularly in oral healthcare, topical antisepsis, and the management of fungal infections such as those caused by *Candida* and dermatophytes. Their ability to target planktonic cells, disrupt biofilms, and suppress virulence factors underscores their relevance in clinical and pharmaceutical contexts. However, challenges remain, such as the variability in chemical composition due to geographical and environmental factors, which can affect efficacy [78,79]. Moreover, while in vitro studies are abundant, more in vivo animal models and human clinical trials are needed to confirm efficacy, determine optimal dosages, and assess safety profiles for therapeutic applications.

### 5.2. Antioxidant and Anti-Inflammatory Activities

The antioxidant and anti-inflammatory properties of *Mentha* EOs are crucial to their health-promoting effects, contributing to their potential in preventing and managing various chronic illnesses. These activities are largely attributed to the presence of phenolic compounds, flavonoids, and specific monoterpenes that can scavenge free radicals, reduce oxidative stress, and modulate inflammatory pathways.

Numerous studies have consistently demonstrated the significant antioxidant potential of *Mentha* EOs. A key component often implicated in the anti-inflammatory effects of *Mentha* EOs, particularly *M. pulegium*, is pulegone. El abdali et al. (2024) [38] assessed the antioxidant capacity of Moroccan *M. pulegium* EO (72.05% pulegone) using FRAP and DPPH assays, reporting notable potential with EC_50_ and IC_50_ values of 26.500 ± 0.200 mg/mL and 54.630 ± 1.350 mg/mL, respectively, along with a total antioxidant capacity of 52.610 ± 4.734 mg AAE/g EO. Cheraif et al. (2020) [40] further supported this, showing *M. pulegium* EO (76.9% pulegone) exhibited DPPH scavenging activity (IC_50_ values ranging from 2.61 to 91.25 mg/mL) and FRAP values (0.97–8.17 µmol Trolox equivalents (TX)/g sample), and ABTS assay values (7.01 to 2.40 µmol TX/g sample). Yang et al. (2019) [74] demonstrated that pulegone inhibited inflammation in LPS-induced sepsis mice. It significantly reduced serum levels of various cytokines (IL-18, IL-1β, IL-5, TNF-α, IFN-γ, MCP-1, MIP-1β, M-CSF, GM-CSF) and decreased the mRNA and protein expression of key components of the NLRP3 inflammasome (ASC, NLRP3, and Caspase-1) in lung tissue.

Comparative studies highlight differences in antioxidant efficacy among *Mentha* species. Wu et al. (2019) [34] analyzed the antioxidant properties of EOs from *M. piperita*, *M. spicata*, and *M. gracilis*. All three EOs demonstrated prominent radical scavenging and Fe3+ reducing activity in chemical-based assays (reducing power, DPPH, TEAC). Notably, *M. piperita* EO had the lowest (*p* < 0.05) half maximal effective concentration (EC_50_) in DPPH and TEAC assays and higher efficacy in the reducing power assay compared to native and Scotch spearmint EOs. All three EOs equivalently mitigated chemical-induced lipid peroxidation in liver tissues in a dose-dependent manner. Furthermore, *M. piperita* and *M. gracilis* EOs significantly increased the survival rate of *C. elegans* under H_2_O_2_-induced oxidative stress, comparable to ascorbic acid, while *M. spicata* EO did not show this protective effect at the same doses. This suggests a superior antioxidant profile for peppermint and Scotch spearmint in certain contexts.

*M. suaveolens* EO has also shown remarkable antioxidant activities. Bouyahya et al. (2019) [44] reported *M. suaveolens* EO (56.28% piperitenone oxide) exhibited significant antioxidant capacity with IC_50_ values of (64.76 ± 2.24) μg/mL (DPPH), (82.73 ± 3.34) μg/mL (ABTS), and (93.35 ± 4.45) μg/mL (FRAP). Al-Mijalli et al. (2022) [45] also found *M. suaveolens* EO to possess remarkable antioxidant properties, though Salvia officinalis EO demonstrated better results in DPPH, ABTS, and FRAP tests in their comparative study. This indicates that while *M. suaveolens* is a potent antioxidant, its efficacy can vary relative to other plant EOs.

The antioxidant capacity is not limited to EOs but also extends to aqueous extracts. Brahmi et al. (2024) [46] found that the aqueous extract of *M. subtomentella* displayed a significantly higher antioxidant capacity (IC_50_ = 0.17 ± 0.01 mg/mL) than its EO (IC_50_ = 13% ± 0.01% *v*/*v*), which exhibited relatively lower activity. This suggests that non-volatile phenolic compounds like rosmarinic acid, identified as a major component in the aqueous extract, contribute substantially to the overall antioxidant profile of the plant. Zouari-Bouassida et al. (2018) [28] similarly found that the ethyl acetate fraction of *M. longifolia* leaves, rich in phenolic and flavonoid compounds, displayed the most active DPPH scavenging ability (IC_50_ = 12.64 μg/mL) and interesting β-carotene bleaching inhibition (IC_50_ = 34.75 μg/mL).

Beyond in vitro assessments, in vivo studies have provided compelling evidence for the antioxidant benefits of *Mentha* EOs. A notable study by W. Zhang et al. (2023) [69] demonstrated that repeated inhalation of peppermint EO improved exercise performance in endurance-trained rats. The mechanism involved extended exhaustion time and, crucially, a reduction in oxidative damage compared to untreated rats. This suggests that peppermint oil can enhance physical endurance by mitigating exercise-induced oxidative stress, a finding with implications for sports nutrition and recovery. In the context of animal health and nutrition, the inclusion of *Mentha* EOs or blends containing them has shown significant antioxidant benefits.

*Mentha* EOs have demonstrated significant anti-inflammatory potential through modulation of various inflammatory mediators. Zouari-Bouassida et al. (2018) [28] provided compelling in vivo evidence for the anti-inflammatory properties of *M. longifolia*. Their study revealed that both tested extracts of *M. longifolia* significantly suppressed carrageenan-induced paw edema (*p* < 0.001) at 5 h post-induction compared to control groups. Remarkably, the *M. longifolia* extracts (200 mg/kg) demonstrated superior efficacy to the standard drug indomethacin, achieving 62.29% inhibition compared to 49.15% for the pharmaceutical agent (*p* < 0.01). Histological analysis confirmed that while the carrageenan-only group exhibited severe edema with substantial inflammatory cell infiltration, both *M. longifolia* extracts and indomethacin pretreatments substantially ameliorated these pathological alterations. The anti-inflammatory mechanism appears to involve cyclooxygenase inhibition, analogous to conventional nonsteroidal anti-inflammatory drugs. Complementing these findings, Messaoudi et al. (2021) [39] confirmed the in vivo anti-inflammatory activities of *M. pulegium* leaf extracts. Oral administration of the aqueous extract to mice with carrageenan-induced hind paw edema produced significant anti-inflammatory effects (*p* < 0.001) at all tested doses. The extract demonstrated substantial reduction in carrageenan-induced paw edema compared to the reference drug diclofenac sodium (10 mg/kg), with edema paw weight decreasing to 22.16% compared to 52.97% inhibition exhibited by the pharmaceutical compound. These pharmacological properties, likely attributable to the presence of phenolic compounds, provide scientific validation for the traditional use of *Mentha* species in managing inflammatory conditions.

A more specific mechanism of anti-inflammatory action was elucidated by Biltekin et al. (2023) [32], who evaluated the in vitro anti-inflammatory effects of *M. spicata* L. EO. They found that *M. spicata* EO (72.8% carvone, 12.6% limonene) showed selective COX-2 inhibition, with a selectivity index (SI) value of 0.67. Cyclooxygenase-2 (COX-2) is an enzyme primarily responsible for producing pro-inflammatory prostaglandins, and its selective inhibition is a desirable target for anti-inflammatory drugs with fewer side effects than non-selective COX inhibitors. This finding suggests a targeted anti-inflammatory mechanism for spearmint EO.

The advantages of *Mentha* EOs in antioxidant and anti-inflammatory applications include their natural origin, multi-target action, and potential to mitigate chronic disease progression. The detailed in vivo studies, particularly those demonstrating reductions in oxidative damage markers and inflammatory cytokines, provide strong support for their therapeutic potential. However, a disadvantage is the variability in chemical composition, which can lead to inconsistent efficacy across different batches or species. More standardized products with defined chemical profiles are needed [80]. Furthermore, while animal models offer valuable insights, comprehensive human clinical trials are still required to establish optimal dosages, long-term safety, and efficacy in various human inflammatory and oxidative stress-related conditions. The integration of *Mentha* EOs into nutritional strategies, such as functional foods or dietary supplements, holds significant promise for promoting health and preventing disease.

### 5.3. Neurological and Digestive Benefits

The intricate connection between the gut and the brain, often referred to as the gut–brain axis, plays a pivotal role in maintaining overall health, influencing everything from mood and cognitive function to nutrient absorption and immune responses [81]. Disruptions in this axis can lead to a myriad of neurological and digestive disorders. *Mentha* EOs, with their traditional use in alleviating gastrointestinal discomfort and their emerging neuroactive properties, are increasingly being recognized for their potential to positively impact both these interconnected systems.

The digestive benefits of *Mentha* EOs are perhaps the most widely recognized and traditionally utilized. *M. piperita* (peppermint) has a long history of use in managing various digestive disorders, including nausea, indigestion, and irritable bowel syndrome (IBS) [70]. Its antispasmodic properties, primarily attributed to menthol, help relax the smooth muscles of the gastrointestinal tract, thereby alleviating cramps and discomfort. While direct human clinical trials on *Mentha* EOs for digestive benefits were not extensively detailed in the provided abstracts, the traditional uses are strongly supported by animal studies and mechanistic insights.

In animal models, *Mentha* EOs have demonstrated significant improvements in gut health and functionality. Chen et al. (2024) [71] investigated the effects of microencapsulated EOs (MEO) supplementation in weaned piglets. The MEO, which include *Mentha* components, increased average daily gain (ADG) and average daily feed intake (ADFI), reduced diarrhea rates, and significantly improved intestinal structure. Specifically, MEO increased the duodenum villus height to crypt depth (V:C) ratio and jejunal villi height, indicating enhanced nutrient absorption and gut integrity. Furthermore, MEO supplementation enhanced appetite and promoted beneficial bacteria diversity in the intestinal microbiome. This study highlights the potential of EOs to act as growth promoters and gut health enhancers in animal nutrition, offering an alternative to antibiotic growth promoters. Similarly, Zhang et al. (2024) [82] in their study on sows, found that oregano EO (OEO) supplementation improved fecal microbiota composition, decreasing the abundance of potentially harmful Proteobacteria and Actinobacteria while increasing beneficial bacteria like Lactobacillus and Prevotellaceae UCG 003 and UCG 005. Although this study focused on oregano, the principles of gut microbiota modulation by EOs are broadly applicable to *Mentha* EOs, which share similar monoterpene profiles and mechanisms of action. The review by Arora & Sharma (2023) [83] specifically explores the role of *Mentha* in gut microbiota, emphasizing its anticholinergic action, ability to block PGE2 and GM1 receptors, and bactericidal, viricidal, and fungicidal properties, which collectively contribute to balancing gut bacteria and managing gut-related diseases. This comprehensive perspective underscores the multifaceted ways *Mentha* herbs interact with the gut microbiome and physiology.

Beyond their established digestive benefits, accumulating evidence points to the neurological advantages of *Mentha* EOs, particularly through their influence on cognitive function and mood. Although the underlying mechanisms require further elucidation, these effects are likely mediated by interactions with neurotransmitter systems, anti-inflammatory actions, and antioxidant protection within the brain. Supporting this, an in vivo study by Susmita et al. (2024) [72] evaluating *Mentha* spp. EO reported marked improvements in cognitive tests, with the treatment group demonstrating a 15% enhancement in memory recall and a 12% increase in attention span. This notable finding provides evidence for the direct nootropic potential of *Mentha* EOs.

Menthol, a primary component of peppermint oil, has also been implicated in neurological effects. Park et al. (2009) [73] investigated menthol’s role in enhancing the antiproliferative activity of 1α,25-dihydroxyvitamin D3 in LNCaP cells. While this study primarily focused on cancer, it revealed that menthol increases intracellular calcium concentration ([Ca^2+^]i), suggesting a potential cross-talk with calcium signaling pathways. Calcium signaling is fundamental to neuronal function, including neurotransmission and synaptic plasticity. Although this study did not directly assess cognitive function, the observed modulation of calcium dynamics by menthol provides a mechanistic basis for potential neuroactive effects. The traditional use of *Mentha* species for neurological functions, as noted by Hafeeza & Kouser (2025) [70], further supports the exploration of these benefits.

The advantages of *Mentha* EOs for neurological and digestive health lie in their natural origin, traditional acceptance, and multi-target mechanisms, including direct antimicrobial action, gut microbiota modulation, anti-inflammatory effects, and potential neuroactive properties. These benefits are particularly relevant in nutritional and medical contexts, offering complementary strategies for managing common conditions. However, a significant disadvantage is the relative scarcity of human clinical trials specifically designed to assess the neurological and digestive benefits of *Mentha* EOs, especially concerning standardized dosages and long-term outcomes. Most of the compelling evidence comes from in vivo animal models, which, while informative, may not always directly translate to human physiology. Future research should prioritize well-designed human clinical trials to validate these benefits, establish optimal therapeutic regimens, and explore the precise molecular mechanisms underlying their effects on the gut–brain axis. The development of targeted delivery systems could also enhance their efficacy and reduce potential side effects.

### 5.4. Respiratory and Cognitive-Enhancing Potential

The therapeutic applications of *Mentha* EOs extend to the respiratory system and cognitive function, areas where their volatile compounds, particularly menthol, can exert direct and indirect beneficial effects. Traditional medicine has long recognized the utility of mints for alleviating respiratory discomfort, and modern research is beginning to uncover the mechanisms behind these observations, alongside their emerging role in cognitive enhancement.

For respiratory health, *M. piperita* (peppermint) EO is a well-known traditional remedy for various respiratory conditions, including congestion, coughs, and colds [70]. The primary active compound, menthol, is responsible for the characteristic cooling sensation and is believed to act as a decongestant and antitussive. While direct human clinical trials specifically on *Mentha* EOs for respiratory conditions were not extensively detailed in the provided abstracts, the traditional uses are widely accepted and supported by the physiological effects of menthol.

An interesting in vivo animal study by Zhang et al. (2023) [69] provides indirect but compelling evidence for the respiratory benefits of peppermint EO. This study demonstrated that repeated inhalation of peppermint EO improved exercise performance in endurance-trained rats. The rats showed an extended exhaustion time and reduced oxidative damage. While the study focused on exercise performance, improved endurance is intrinsically linked to enhanced respiratory efficiency and oxygen utilization. The inhalation route of administration is particularly relevant for respiratory applications, suggesting that the volatile components of peppermint oil can exert systemic effects, including those that support respiratory function during physical exertion. This finding has implications for sports medicine and general wellness, indicating that peppermint oil inhalation could be a natural aid for improving physical stamina and recovery by optimizing physiological responses, potentially including respiratory capacity.

In the context of animal health, EOs, including those from *Mentha* species, are being explored for their potential to mitigate respiratory diseases. Magossi et al. (2024) [84] conducted a pilot study evaluating a single intranasal dose of an EO spray in feedlot cattle. This spray, containing a blend of five EOs (though specific *Mentha* species were not named, the context of EOs for respiratory health is relevant), conferred modulation of the nasopharyngeal microbiota and short-term inhibition of Mannheimia, a key pathogen in bovine respiratory disease (BRD). The intranasal administration method directly targets the respiratory tract, demonstrating a localized effect on pathogens and microbiota. While this study did not specifically use *Mentha* EOs, it highlights the feasibility and potential of EO-based interventions for respiratory health in animals, a field where *Mentha* EOs could certainly play a role given their known properties.

Regarding cognitive-enhancing potential, the *Mentha* genus has shown promising effects, particularly in human studies. The in vivo and human clinical trial data from Susmita et al. (2024) [72] reported that *Mentha* spp. oil led to significant improvements in cognitive function. Specifically, memory recall improved by 15% and attention span by 12% in the treatment group. These are direct measures of cognitive enhancement in humans, providing strong evidence for the neurocognitive benefits of *Mentha* EOs. This aligns with the traditional recognition of *Mentha* species for supporting neurological functions, as mentioned by Hafeeza & Kouser (2025) [70]. The mechanisms underlying these cognitive effects are likely multifaceted, potentially involving modulation of neurotransmitter systems, reduction in neuroinflammation, and antioxidant protection of neuronal cells, as discussed in the context of neurological benefits in the previous section. The volatile nature of EOs allows for rapid absorption through the olfactory system and lungs, enabling direct access to the brain and potentially influencing mood, alertness, and cognitive processes.

The advantages of *Mentha* EOs for respiratory and cognitive enhancement include their natural origin, pleasant aroma, and the non-invasive nature of inhalation as a delivery method. The human clinical data on cognitive improvement is particularly compelling, offering a tangible benefit for daily life and potentially for age-related cognitive decline. For respiratory health, the traditional uses are strong, and the indirect evidence from exercise performance in animals suggests physiological benefits. However, a disadvantage is the relative lack of dedicated human clinical trials specifically investigating the direct effects of *Mentha* EOs on various respiratory conditions, such as asthma or chronic obstructive pulmonary disease (COPD). More targeted research is needed to establish efficacy, optimal dosages, and safety profiles for these specific applications. Similarly, while the cognitive benefits are promising, further large-scale, placebo-controlled human trials are warranted to confirm these effects and elucidate the underlying neurobiological mechanisms. Integrating *Mentha* EOs into aromatherapy practices or as dietary supplements could offer natural avenues for supporting respiratory wellness and cognitive vitality.

### 5.5. Emerging Anticancer Properties

The quest for novel, effective, and less toxic anticancer agents has led researchers to explore natural products, with EOs emerging as a promising avenue [85,86,87]. *Mentha* EOs, known for their diverse biological activities, are increasingly being investigated for their potential anticancer properties, with studies revealing their ability to inhibit cancer cell proliferation, induce apoptosis, and modulate key signaling pathways involved in carcinogenesis. While this area of research is still in its nascent stages, particularly concerning human clinical trials, compelling evidence from in vitro and in vivo animal models suggests a significant therapeutic potential.

Several *Mentha* species and their key components have demonstrated anticancer effects against various cancer cell lines. Biltekin et al. (2023) [32] evaluated the in vitro anti-inflammatory and anticancer effects of *M. spicata* L. EO. The oil, rich in carvone (72.8%), limonene (12.6%), and 1,8-cineole (2.2%), exhibited significant cytotoxic effects on human lung adenocarcinoma (A549), breast adenocarcinoma (MCF7), and prostate cancer (PC3) cells, with IC50 values of 672.13 ± 2.57, 708.27 ± 2.05, and 206.49 ± 1.48 μg/mL, respectively. Crucially, it showed no cytotoxic effects on healthy HEK293 cells, indicating a degree of selectivity. The study also found that *M. spicata* EO significantly increased apoptosis in these cancer cell lines and selectively inhibited COX-2, an enzyme often overexpressed in various cancers and linked to inflammation and tumor progression. This dual mode of action—inducing apoptosis and inhibiting COX-2—is a novel and important finding, suggesting that *M. spicata* EO could target multiple pathways involved in cancer development.

*M. longifolia* EO has also shown direct anticancer activity. Afkar & Somaghian (2024) [30] reported that *M. longifolia* EO from ecotype 3 significantly reduced the viability of SW742 tumor cells. While the specific mechanism was not detailed in the abstract, this finding provides direct evidence of its cytotoxic potential against cancer cells. The chemical composition of *M. longifolia* EO, often characterized by pulegone and other monoterpenes, likely contributes to these effects [88,89].

Menthol, a major component of *M. piperita*, has been investigated for its role in cancer therapy. Park et al. (2009) [73] demonstrated that menthol enhances the antiproliferative activity of 1α,25-dihydroxyvitamin D3 in LNCaP prostate cancer cells. Although menthol alone lacked significant antiproliferative activity, its combination with 1α,25-dihydroxyvitamin D3 boosted growth inhibition. Western blot analysis showed that this combination modulated the expression of bcl-2 (an anti-apoptotic protein) and p21 (a cell cycle inhibitor), suggesting that menthol could enhance the therapeutic effects of other anticancer agents by influencing cell cycle progression and apoptosis. This highlights the potential for *Mentha* components to act as chemosensitizers, improving the efficacy of existing cancer treatments.

The broader potential of *Mentha* species in prostate cancer treatment has been recognized in recent reviews. Pimentel et al. (2024) [90], in a review of therapeutic effects of EOs on prostate cancer, specifically included *Mentha* species among those exhibiting anti-prostate cancer activity. This review noted that EOs from various plants, including *Mentha*, inhibit cell growth, migration, and angiogenesis, and induce apoptosis. While the challenges of lipophilicity, instability, and volatility of EOs are acknowledged, the review suggests that processing EOs into pharmaceutical formulations could address these issues and lead to innovative drug developments.

Pulegone, a prominent monoterpene found in *M. pulegium* and *M. longifolia*, has also been linked to anticancer potential, albeit indirectly through its anti-inflammatory actions. As discussed in the anti-inflammatory section, pulegone inhibits inflammation via suppression of the NLRP3 inflammasome and reduction in cytokine production [74], and regulates NF-κB and Nrf-2 signaling pathways [91]. Given that chronic inflammation is a known driver of carcinogenesis, the anti-inflammatory properties of pulegone could contribute to cancer prevention and therapy by mitigating the inflammatory microenvironment that supports tumor growth. While not a direct cytotoxic effect, this indirect mechanism is highly relevant to the broader anticancer potential of *Mentha* EOs.

The mechanisms by which *Mentha* EOs and their components exert anticancer effects are diverse and often involve multiple cellular pathways. These include: (1) Induction of apoptosis: As seen with *M. spicata* EO, which significantly increased apoptosis in cancer cells [32], and menthol’s modulation of bcl-2 expression [73]. Apoptosis, or programmed cell death, is a crucial mechanism for eliminating cancer cells. (2) Inhibition of cell proliferation: Demonstrated by the reduction in tumor cell viability by M. spicata and *M. longifolia* EOs [30,32]. (3) Modulation of inflammatory pathways: Selective inhibition of COX-2 by *M. spicata* EO [32] and the broader anti-inflammatory effects of pulegone on NF-κB and NLRP3 inflammasome pathways [74] are critical, as inflammation fuels cancer progression. (4) Synergistic effects with other agents: Menthol’s ability to enhance the activity of vitamin D3 suggests that *Mentha* components could be used in combination therapies to improve outcomes and potentially reduce the dosage of conventional chemotherapeutic drugs [73].

The advantages of *Mentha* EOs in anticancer research include their natural origin, multi-target action, and potential for fewer side effects compared to conventional chemotherapy. The evidence from in vitro studies is robust, demonstrating direct cytotoxicity, apoptosis induction, and modulation of key cancer-related pathways. However, a significant disadvantage is the early stage of this research. Most findings are from in vitro cell line studies, with limited in vivo animal models and virtually no human clinical trials specifically on *Mentha* EOs for cancer treatment. This gap needs to be addressed to translate these promising laboratory findings into clinical applications. Challenges such as bioavailability, stability, and potential toxicity at high concentrations also need careful consideration. Future research should focus on: (1) conducting more in vivo animal studies to confirm efficacy and safety, (2) elucidating the precise molecular mechanisms of action, (3) developing advanced delivery systems to enhance bioavailability and targeted delivery, and (4) exploring synergistic combinations with conventional anticancer drugs to improve therapeutic outcomes and reduce adverse effects. The identification of specific bioactive compounds and their optimal concentrations will be crucial for developing *Mentha*-derived anticancer agents.

## 6. Challenges and Future Perspectives

The promising health-promoting activities and industrial applications of *Mentha* EOs are tempered by several significant challenges. Addressing these hurdles through interdisciplinary research and technological innovation is crucial for unlocking their full potential and ensuring their safe and effective integration into pharmaceuticals, food, and cosmetics.

### 6.1. Standardization and Quality Control Issues

A primary impediment to the reliable application of *Mentha* EOs is their inherent chemical variability, which directly influences their therapeutic efficacy, aroma profile, and safety. As detailed in Section 3.2, this compositional fluctuation stems from a complex interplay of genetic background, geographical origin, environmental conditions (e.g., climate, water quality, soil), harvest timing, and extraction methodologies [2,9,14,16,28,47,48]. Such inconsistency poses a major challenge for industrial standardization, a prerequisite for pharmaceutical applications and consistent product quality in the food and cosmetic sectors.

Recent studies underscore this variability. For instance, Mustafa et al. (2024) [16] documented substantial differences in oil composition across 20 distinct *M. longifolia* accessions, attributing these differences to both genetic and cultivation factors. Similarly, Zouari-Bouassida et al. (2018) [28] demonstrated that the proportions of major constituents in *M. longifolia* shift with the seasons, highlighting the critical impact of harvest time. Furthermore, Haddou et al. (2023) [14] revealed that even the quality of irrigation water can significantly alter the chemical profile of *M. piperita* oil, indicating that consistent output requires rigorous control over both agricultural and processing parameters.

This lack of standardization leads to several critical issues: fluctuating levels of active compounds result in unreliable biological performance; the presence of potentially toxic components like pulegone in *M. pulegium* raises safety concerns [13,38]; and the high economic value of EOs creates a vulnerability to adulteration with synthetic compounds or inferior oils [59]. Taylan et al. (2021) [59] exposed this risk by using ATR-FTIR spectroscopy to detect adulteration in commercial *Mentha* EOs, emphasizing the need for robust authentication techniques.

Future efforts must therefore focus on a multi-pronged approach: leveraging analytical tools like GC-MS and GC-FID to establish comprehensive chemical fingerprints [34,38,39]; developing and enforcing rigorous quality standards and pharmacopeial monographs for specific chemotypes; implementing stringent supply chain controls from cultivation to final product; and utilizing genomic insights [17,18,19] for marker-assisted breeding of stable, high-yielding cultivars with consistent chemical profiles. Only through such measures can the industry ensure consumer safety, meet regulatory requirements, and deliver *Mentha* EOs with predictable and dependable therapeutic effects.

### 6.2. Bioavailability and Formulation Advances

The promising bioactivities of *Mentha* EOs are often hampered by their inherent physicochemical limitations, including high volatility, poor aqueous solubility, and susceptibility to degradation (oxidation, light, heat) [2,3,9,48,92,93]. These drawbacks lead to low bioavailability, challenges in formulating stable aqueous-based products for food, pharmaceutical, and cosmetic use, and ultimately, diminished efficacy. The hydrophobic nature of EOs impedes their absorption and uniform dispersion, while their chemical instability can lead to the degradation of active monoterpenes, resulting in loss of potency and potential formation of undesirable off-odors or toxic byproducts [48]. These issues are particularly acute in oral applications, where gastrointestinal degradation further limits systemic absorption [3].

Advanced encapsulation technologies offer powerful solutions to overcome these barriers. Nanoemulsions, with droplet sizes between 20 and 200 nm, significantly enhance water dispersibility, stability against degradation, and cellular absorption. Oliveira Filho et al. (2023) [37] demonstrated that nanoencapsulated *M. spicata* oil effectively functioned as a natural preservative, reducing weight loss and fungal decay in papayas. Similarly, Omidian et al. (2025) [92] provided a comprehensive analysis of how nanoemulsions can be engineered to optimize EO performance. Microencapsulation, using polymer matrices to create protective microcapsules (1–1000 µm), shields EOs from environmental stressors and enables controlled release. Fernandes et al. (2024) [93] reviewed the industrial-scale application of spray-drying microencapsulation in foods, where it serves as a natural alternative to synthetic preservatives in products like baked goods and processed meats.

On a smaller scale, nanoencapsulation strategies—including nanoliposomes and polymeric nanoparticles (<100 nm)—offer superior stability, bioavailability, and potential for targeted delivery. Their efficacy has been showcased in areas such as poultry feed [3] and eco-friendly biopesticides [9]. These advanced delivery systems confer multiple advantages: prolonged shelf-life by protecting labile compounds, enhanced bioavailability due to increased surface area and improved absorption, controlled release kinetics for sustained activity, and the ability to mask strong odors.

Future research should prioritize the development of biodegradable and food-grade encapsulation materials, refinement of sustainable and scalable synthesis methods [2,53], rigorous in vivo studies to confirm the safety and efficacy of novel formulations, and the establishment of clear regulatory frameworks for nanoformulations. Overcoming the bioavailability and stability challenges is a pivotal step toward realizing the full commercial and therapeutic potential of *Mentha* EOs.

### 6.3. Safety Assessment and Toxicity Profiling

The “all-natural” label often associated with EOs can foster a misleading perception of absolute safety, which may lead to improper use and associated health risks. A comprehensive and scientifically grounded safety assessment is therefore imperative, especially as *Mentha* EOs are incorporated into medicinal and wellness products. Toxicity is intrinsically linked to chemical composition, which can vary dramatically between species and even batches. A prominent example is *M. pulegium* EO, characterized by high concentrations of pulegone, a compound with documented hepatotoxic potential at elevated doses [13,38,39,40]. Studies by El abdali et al. (2024) [38], Piras et al. (2019) and Messaoudi et al. (2021) [13,39] consistently report pulegone as the dominant component, frequently exceeding 70% of the oil, necessitating cautious use and clear labeling, particularly regarding internal consumption. As cautioned by Tsitlakidou et al. (2023) [2], the misconception of inherent safety can result in dermal irritation, allergic reactions, or adverse drug interactions—risks that also extend to more common varieties like *M. piperita* or *M. spicata*.

A thorough toxicity profile must consider multiple factors: dose–response relationships, compound-specific hazards, route of administration (oral, dermal, inhalation), individual susceptibility, and potential interactions with pharmaceuticals. Future research must prioritize rigorous in vitro and in vivo toxicological studies of various *Mentha* EOs and their key constituents, encompassing acute, subchronic, and reproductive toxicity assessments. In silico approaches, such as the PASS algorithm, ADME modeling, and Pro-Tox II utilized by El abdali et al. (2024) [38] and Haddou et al. (2023) [14], provide valuable preliminary safety screens and can guide focused experimental work.

As highlighted by Al-Mijalli et al. (2022) [45] and Nehme et al. (2021) [94], well-designed clinical trials and post-market surveillance are indispensable for establishing safe human dosage ranges and confirming efficacy. Regulatory bodies play a critical role in establishing clear guidelines on maximum allowable concentrations of specific compounds, standardized labeling requirements, and usage restrictions. Concurrently, public education initiatives are essential to dispel the myth of absolute safety and to promote the informed and responsible use of *Mentha* EOs among consumers and healthcare practitioners alike.

### 6.4. Interdisciplinary Research on Mentha EOs

The multifaceted nature of *Mentha* EOs—spanning their complex chemistry, intricate biosynthesis, diverse biological activities, and practical applications—demands a concerted interdisciplinary research approach to address existing challenges. Integrating expertise from plant biology, genomics, phytochemistry, pharmacology, materials science, and agronomy is key to unlocking their full potential.

Cutting-edge genomic resources, such as the haplotype-resolved genome assembly for *M. suaveolens* [17], enable the identification of genetic markers linked to desirable traits, paving the way for precision breeding of cultivars with stable, high-value chemotypes. Concurrently, breakthroughs in elucidating biosynthetic pathways [18] and understanding epigenetic regulation [19] offer promising strategies for enhancing the yield of target monoterpenes through metabolic engineering.

Cross-disciplinary collaboration is equally vital for developing sustainable agricultural products. The demonstrated efficacy of *Mentha* EOs against a range of pests—including termites [95], aphids [11], plant pathogens [30,96], and fungi [16,61]—underscores their potential as eco-friendly biopesticides. Realizing this potential requires entomologists, pathologists, and chemists to work together to identify the most effective chemotypes, decipher their modes of action, and develop stable, field-ready formulations. Furthermore, combining EOs with biocontrol agents like Beauveria bassiana [11] could advance integrated pest management strategies. Similarly, the replacement of antibiotic growth promoters with EOs in animal feed [3,48,94] necessitates input from nutritionists, veterinarians, and formulation scientists to ensure palatability, bioavailability, efficacy, and minimal risk of contributing to antimicrobial resistance.

Innovations in delivery systems represent another critical frontier. Nanotechnology and microencapsulation [3,92,93,97,98] hold the key to overcoming challenges of volatility, poor solubility, and degradation—a task requiring close collaboration between materials scientists and pharmaceutical researchers. Rigorous investigations into release kinetics, stability under various conditions, and in vivo bioavailability are essential for translating laboratory successes into real-world applications. The synergistic use of Carnauba wax nanoemulsions and *M. spicata* oil to extend fruit shelf-life [37] exemplifies the power of such interdisciplinary problem-solving.

Finally, robust pharmacological and clinical validation is paramount. While in vitro studies have compellingly demonstrated antimicrobial, antioxidant, anti-inflammatory, and anticancer properties [13,28,30,31,32,34,38], in vivo trials and clinical research are needed to elucidate mechanisms of action, establish dose–response relationships, and confirm long-term safety profiles. As noted by Wei et al. (2023) [99], optimizing therapeutic applications hinges on a deeper understanding of metabolite interactions. Through coordinated, interdisciplinary efforts, researchers can standardize formulations, enhance efficacy, and ensure the safe and sustainable integration of *Mentha* EOs across various industries, from agriculture to medicine.

To effectively translate laboratory findings into tangible products, future interdisciplinary efforts should be strategically directed. In pharmaceutical development, collaboration between phytochemists, pharmacologists, and clinical researchers is essential to validate the efficacy of specific *Mentha* chemotypes in targeted human trials, such as using menthol-rich oils for respiratory relief or carvone-dominated oils in oral care products. Simultaneously, material scientists and nanotechnologists must work on optimizing nano-delivery systems to enhance the stability, bioavailability, and targeted delivery of these volatile compounds.

For agri-food applications, partnerships among food scientists, plant biologists, and agricultural engineers are key. Research should focus on integrating selected *Mentha* EOs into edible films and coatings for specific high-value fruits and vegetables, leveraging their antifungal and antioxidant properties identified in studies on papaya and other models. In animal husbandry, nutritionists and veterinarians should conduct large-scale trials to determine the optimal inclusion levels of *Mentha* EOs in feed formulations to improve growth performance, gut health, and disease resistance in livestock, thereby providing viable alternatives to antibiotic growth promoters. By fostering such targeted, application-oriented collaborations, the full potential of *Mentha* EOs can be realized across diverse and sustainable industrial sectors.

## Figures and Tables

**Table 1:** Comprehensive Summary of Health-Beneficial Effects of *Mentha* EOs and Extracts.

Species	Pharmacological Activity	Study Type	References
*M. piperita*	Antimicrobial/Antifungal	In vitro	[35]
		In vitro	[11]
	Antioxidant	In vitro	[34]
		In vivo (Animal)	[69]
		In vivo (Animal)	[34]
	Digestive Benefits	Review/Mechanism	[70]
		In vivo (Animal)	[71]
	Neurological/Cognitive	In vivo (Human)	[72]
	Respiratory Benefits	Review/Mechanism	[70]
		In vivo (Animal)	[69]
	Anticancer	In vitro	[73]
*M. spicata*	Antimicrobial/Antifungal	In vitro	[13,23]
	Antioxidant	In vitro	[34]
	Anti-inflammatory	In vitro	[32]
	Anticancer	In vitro	[32]
*M. pulegium*	Antimicrobial/Antifungal	In vitro	[38,39]
	Antioxidant	In vitro	[28,38]
	Anti-inflammatory	In vivo (Animal)	[39]
		In vivo (Animal)	[74]
*M. longifolia*	Antimicrobial	In vitro	[45]
	Antioxidant	In vitro	[28]
	Anti-inflammatory	In vivo (Animal)	[28]
	Anticancer	In vitro	[30]
*M. suaveolens*	Antimicrobial	In vitro	[44]
	Antioxidant	In vitro	[44,45]
*M. subtomentella*	Antioxidant	In vitro	[46]
*M. gracilis*	Antioxidant	In vivo (Animal)	[34]

## Data Availability

No new data were created or analyzed in this study. Data sharing is not applicable to this article.

## Supplementary Materials

The following supporting information can be downloaded at: https://www.mdpi.com/article/10.3390/nu17203258/s1, Figure S1: Monoterpenoid Biosynthesis via the Plastidial MEP Pathway; Figure S2: Structures of Additional Characteristic Monoterpenoids in *Mentha* Essential Oils.

## Abbreviations

The following abbreviations are used in this manuscript:

EO	Essential Oil
GC-MS	Gas Chromatography–Mass Spectrometry
GC-FID	Gas Chromatography with Flame Ionization Detection
ATR-FTIR	Attenuated Total Reflectance–Fourier Transform Infrared Spectroscopy
MEP	Methylerythritol Phosphate Pathway
IPP	Isopentenyl Pyrophosphate
DMAPP	Dimethylallyl Pyrophosphate
GPP	Geranyl Pyrophosphate
GPPS	Geranyl Pyrophosphate Synthase
LS	Limonene Synthase
L6OH	Limonene 6-Hydroxylase
L3OH	Limonene 3-Hydroxylase
CD	Carveol Dehydrogenase
ISPD	Isopiperitenol Dehydrogenase
ISPR	Isopiperitenone Reductase
ISPI	Isopulegone Isomerase
MFS	Menthofuran Synthase
PR	Pulegone Reductase
MD	Menthol Dehydrogenase
TPS	Terpene Synthase
CYP450	Cytochrome P450 Monooxygenase
TF	Transcription Factor
MeJA	Methyl Jasmonate
JA	Jasmonic Acid
DEG	Differentially Expressed Gene
KEGG	Kyoto Encyclopedia of Genes and Genomes
WGCNA	Weighted Gene Co-expression Network Analysis
PGT	Peltate Glandular Trichome
ADG	Average Daily Gain
ADFI	Average Daily Feed Intake
V:C	Villus Height to Crypt Depth Ratio
OEO	Oregano Essential Oil
BRD	Bovine Respiratory Disease
COPD	Chronic Obstructive Pulmonary Disease
MIC	Minimum Inhibitory Concentration
MBC	Minimum Bactericidal Concentration
FRAP	Ferric Reducing Antioxidant Power
DPPH	2,2-Diphenyl-1-picrylhydrazyl
ABTS	2,2′-Azino-bis(3-ethylbenzothiazoline-6-sulfonic acid)
TEAC	Trolox Equivalent Antioxidant Capacity
COX	Cyclooxygenase
NLRP3	NOD-, LRR- and pyrin domain-containing protein 3
IL	Interleukin
TNF-α	Tumor Necrosis Factor-alpha
IFN-γ	Interferon-gamma
MCP-1	Monocyte Chemoattractant Protein-1
MIP-1β	Macrophage Inflammatory Protein-1β
M-CSF	Macrophage Colony-Stimulating Factor
GM-CSF	Granulocyte-Macrophage Colony-Stimulating Factor
NF-κB	Nuclear Factor Kappa B
Nrf-2	Nuclear Factor Erythroid 2-Related Factor 2
IBS	Irritable Bowel Syndrome
MEO	Microencapsulated Essential Oil
ADME	Absorption, Distribution, Metabolism, Excretion
PASS	Prediction of Activity Spectra for Substances
PLSR	Partial Least Squares Regression
PCR	Principal Component Regression
HCA	Hierarchical Cluster Analysis
PCA	Principal Component Analysis
