# Supplementary material for: Mentha Essential Oils: Unraveling Chemotype-Dependent Biosynthesis and Assessing Evidence for Health-Promoting Activities
Source: Nutrients. 2025 Oct 16;17(20):3258. doi: 10.3390/nu17203258 (PMC12567415; doi:10.3390/nu17203258)
Supplement: Supplementary file 1 [file nutrients-17-03258-s001.zip › nutrients-3902123-supplementary.pdf]

nutrients

MDPI

Review

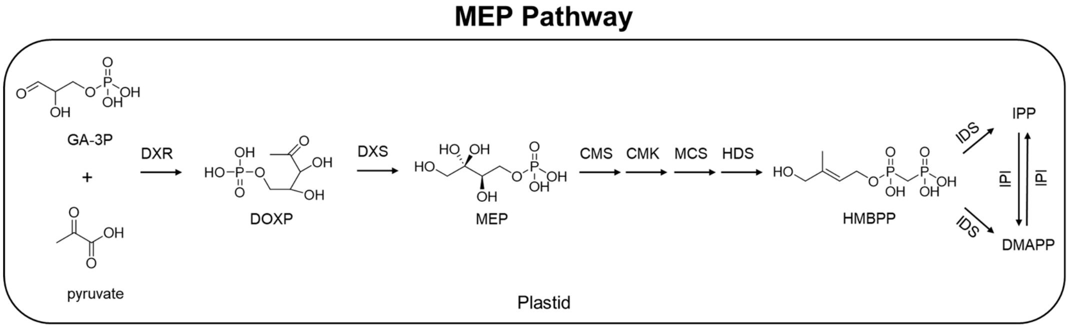

Figure S1. Monoterpenoid Biosynthesis via the Plastidial MEP Pathway. The enzymatic reaction steps are catalyzed by geranyl-1-deoxy-D-xylulose-5-phosphate reductor isomerase (DXR), 1-deoxy-D-xylulose-5-phosphate synthase (DXS), 4- diphosphocytidyl-2C-methyl-D-erythritol-4-phosphate synthase (CMS), 4- diphosphocytidyl-2C-methyl-D-erythritol kinase (CMK), 2C-methyl-D-erythritol 2, 4-diphosphate synthase (MCS), 1-hydroxy-2-methyl-bu- tenyl-4-diphosphate synthase (HDS), IPP/DMAPP synthase (IDS) and isopentenyl diphosphate isomerase (IPI).

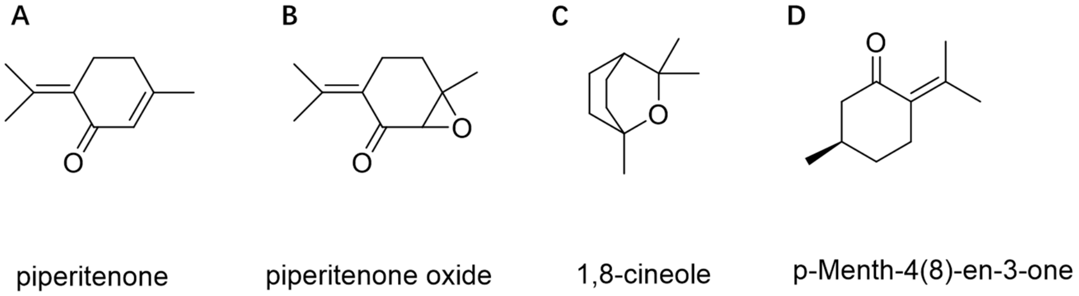

Figure S2. Structures of Additional Characteristic Monoterpenoids in *Mentha* Essential Oils.
